# Drug interactions in elderly (Amirkola Health and Ageing Project)

**DOI:** 10.22088/cjim.13.4.786

**Published:** 2022

**Authors:** Zahra Fath Tabar, Ahmad Reza Moghadamnia, Ali Bijani, Seyed Reza Hosseini, Ali Akbar Moghadamnia

**Affiliations:** 1 Student Research Committee, Babol University of Medical Sciences, Babol, Iran; 2 Social Determinants of Health Research Center, Health Research Institute, Babol University of Medical Sciences, Babol, Iran; 3 Department of Pharmacology and Toxicology, Babol University of Medical Sciences, Babol, Iran

**Keywords:** Elderly, Drug interaction, Inappropriate medication, Inappropriate Drug

## Abstract

**Background:**

Due to physiological changes and co-existing chronic diseases, the elderly has to take various drugs with different mechanisms that may increase the risk of drug interactions and side effects of medications. This study was performed to evaluate the profile of drug interactions of Amirkola elderly patients.

**Methods:**

This cross-sectional descriptive-analytical study is part of the Amirkola Health and Ageing Project (AHAP) which was done during 2012-2013 (Amirkola, Babol, Iran). Initial data collection was done on 1616 persons of ages 60 and older by observing their prescribed drugs and those prepared by self-medications.

**Results:**

Drug interactions were detected in 31.7% (95% CI; 29.41, 33.95) of the drug prescriptions. This included 28% of mild, 63.3% of moderate and 8.7% of severe drug interactions. Cardiovascular drugs (64.4%) were the most frequent drugs that induced drug-interactions. According on Beer criteria 2015, 39.97% of the elderly medications were identified as “inappropriate medication”. NSAIDs had the highest prevalence of inappropriate drugs. There was a significant relationship between female gender, having underlying disease, living alone, having insurance, and polypharmacy with obtained drug interaction results (p<0.05).

**Conclusion:**

The findings of the present study indicate considerable drug interactions among the elderly in Amirkola, which highlights the need for careful prescribing and using of drugs in the elderly.

Increasing population longevity and aging population is one of the major health challenges of the 21st century (1). According to the last census report of the Statistical Center of Iran, the country's population over 60 years of age is estimated to exceed 10 percent in 2021, which is expected to exceed 20 percent by 2050 (2). Increasing age is one of the most complex biological phenomena that results from multiple physiological changes in tissues and increases the likelihood of multiple chronic diseases (3). The presence of these conditions has made the elderly the largest group of drug users in various societies (4). On the other hand, the increasing production of prescription or over-the-counter drugs has led most elders to take more medication than ever before (5). Drug interactions are important cases of medication errors and one of the major challenges in the treatment of patients. In other words, drug interaction is a condition where the efficacy of one drug may be affected by other drugs used concurrently. It can be defined in both pharmacokinetics and pharmacodynamics (6). Although not all drug interactions are predictable, sufficient knowledge of doctors for the potential and the risk factors that increase the likelihood of drug interactions and their understanding of the mechanisms of drug interactions can reduce the rate of actual drug interactions in hospitalized patients (6).

Bogetti *et al.* (2016) in a cross-sectional study in Mexico showed 181 elderly patients with dementia (57 (31.5%) males and 124 (68.5%) females, mean age 80.11±8.28), 107 (59.1%) had potential drug interactions, of which 64 (59.81%) had severe drug interactions. The most common severe drug interactions were citalopram-anti platelet agents (11.6%), clopidogrel-omeprazole (6.1%) and clopidogrel-aspirin (5.5%). Depression, severe dementia along with polypharmacy were identified as having significant effects on severe drug interference (7). 

Appropriate medication for the elderly should be selected based on a history of illness, drug resistance, physical and mental health, physical ability, memory, family support, and so on. Beers criteria can be used to select the right drug. It is used to determine the inappropriate medication in the elderly, which includes three categories: selection of inappropriate medication, inappropriate dose, and inappropriate medication for the underlying disease. According to the criteria, previous studies have shown that many elderly people are exposed to inappropriate medications and their complications (8).

In a study based on the Beers criteria on 676 hospitalized elderly Indian patients, at least one inappropriate drug was found in 87.3%. Metoclopramide, alprazolam, diazepam, digoxin and diclofenac were the most common prescription drugs. NSAIDs are common drugs involved in drug complications and drug interactions in the elderly with cardiovascular diseases (9). Drug interactions impose huge costs on society each year. The Kwan`s study was one of the important and limited investigations that attempted to evaluate economic outcomes of drug interactions. The study estimated that it will indirectly save $29,250 annually if 1% of clinically significant drug interactions were prevented. If the patient develops such drug interactions, it may be added to her/his hospital stay for up to three days. This includes one day for drug interaction detection and two days for decision making, problem solving, and patient return to normal situation (10). Investigating polypharmacy in the elderly is an important issue that needs attention. It is one of the most important issues of health and care for this age group. Polypharmacy has been shown to be common in elderly patients in our country (11, 12), which has led to numerous complications and widespread drug interactions among them. Due to the high importance of drug interactions in the elderly and the lack of comprehensive population-based findings in this area, this study has aimed to determine the rate of drug interactions in the elderly in Amirkola (northern Iran).

## Methods

The present study was a part of the Amirkola Health and Aging Project (AHAP), which started in 2011 as a cohort study on all people 60 years and older in Amirkola (Babol. Iran) (13). This study was approved by the Research Ethics Committee of Babol University of Medical Sciences (Project code: 1394.3160). The city of Amirkola has two health care centers that have the list of all the elderly. All older people were invited to participate in the study. Required information about the project was provided by telephone and home visits. Out of 2234 elderly people, 1616 (72.3%) participated in this study. Data were collected by a trained person using a standard questionnaire. 

The information of medication, including the number, type and duration of drug consumption were collected through patients` self-declared and observing patient's medications by the investigator. These included over-the-counter (OTC or non-prescription) and prescription drugs. To determine the interaction between the drugs which had been prescribed, individuals were first categorized according to the type of disease and the specific drugs of the disease. Then the number of drugs in each category was extracted according to each individual in that category. Drug interactions were then extracted and reported using authentic sources of drug interactions, including drug facts and comparisons or online sources such as webmd.com. Medicines were also categorized according to the Beers 2015 criteria, a measure of detection of inappropriate medication for the elderly developed by the American Society of Elderly (14). conditions for inappropriate medication in elderly have been defined, which include three categories: inappropriate drug selection, inappropriate dose and inappropriate medication for the underlying disease. Drug interactions are also categorized and reported in terms of severity and likelihood of occurrence. Chi-square, t-test and logistic regression were used to analyze the data. Statistical significance between data was considered at p<0.05.

## Results

The number of participants in this study was 1616 people aged 60 years and older, including 883 men and 733 women. Most of the age group (573 participants) belonged to people aged 60-64 years. The majority of the elderly population (1045, 65.8 %) in this study were uneducated. Of all participants, 1434 (88.7%) of the elderly had at least one underlying disease. Most of these people (1508, 93.3%) lived with their families and 1378 were married. Most of these elderly people (82.8%) received their insurance coverage. Overall, drug interactions were seen in 31.7% of the participants (95% CI; 29.41, 33.95). Results showed that moderate drug interactions with 445 (63.3%) were the most frequent. Also, mild drug interactions included 196 (28%) and severe drug interactions 61 (8.7%). Because some prescriptions include combined three types of drug interactions, the total number of drug interactions is greater than number of prescriptions. Among the severe drug interactions, the combination of aspirin-enalapril and atenolol-propranolol each was observed 16 times in 1616 patients. Some of the most important drug interactions are shown in table 1. 

**Table 1.** Most common moderate drug interactions in elderly (Cohort study of Amirkola, AHAP, 2011-2012)

**No.**	**Drug Combination**	**Frequency(%)**
1	Aspirin-Atenolol	50 (11)
2	Aspirin-Metoprolol	40 (8.9)
3	Aspirin-Losartan	30 (6.7)
4	Atenolol-Diclofenac	30 (6.7)
5	Metoprolol-Losartan	25 (5.6)
6	Atenolol-Losartan	20 (4.5)
7	Atorvastatin-Glyburide	18 (4)
8	Propranolol-Glyburide	15 (3.3)
9	Aspirin-Carvedilol	10 (2.2)

According to table 1, among the moderate drug interactions, the combination of aspirin-atenolol 50 times and aspirin-metoprolol 40 times were observed. Losartan-aspirin and diclofenac-atenolol were both seen in 30 (6.7%) patients. Among the mild drug interactions, aspirin-glibenclamide 50 (25.5%), aspirin-triamterene with hydrochlorothiazide 30 (15.3%) and omeprazole-clidinium C 25 (12.7%) were the most frequent. Based on Beers 2015 criteria, 646 prescription drugs for the elderly are considered inappropriate, with the highest number of NSAIDs (245, 15%). Drugs such as fluorazepam, amiodarone, dipyridamole, and ticlopidine were found in only one elderly patient (table 2).

Among the non-steroidal anti-inflammatory drugs, diclofenac was used in 135 (9%) of the elderly. Glibenclamide was also used in 228 (14.25%) of the elderly. There was a significant relationship between gender, having underlying disease, living alone, having insurance, and polypharmacy with drug interactions (p<0.05). According to the results, drug interactions were higher in women, in patients with various underlying diseases, living alone and singles, those with insurance, and those with polypharmacy (more than 4 drugs). But there was no significant difference between age groups and education level. Among the variables, ORs (odds ratio) of underlying disease was 100.207 (95% CI; 13.998, 717.360) and polypharmacy 17.262 (95% CI; 12.954, 23.004), respectively, which showed the most prevalent among the variables affecting drug interactions (table 3). 

**Table 2:** Frequency (%) of inappropriate drugs according Beers 2015 criteria, study on drug interactions in prescriptions for elderly patients (Cohort study of Amirkola, AHAP, 2011-2012)

**Drug Group**	**Inappropriate Drug**	**Frequency(%)**
NSAIDs	Diclofenac	145 (9)
	Ibuprofen	24 (1.5)
	Mefenamic Acid	3 (0.18)
	Naproxen	18 (1.1)
	Piroxicam	5 (0.3)
	Indomethacin	50 (3.1)
	Total	245 (15)
Antidepressants	Amitriptyline	23 (1.4)
	Nortriptyline	17 (1.06)
	Imipramine	11 (0.68)
	Trimipramine	2 (0.12)
	Doxepin	2 (0.12)
	Total	55 (3.4)
Sedative-Hypnotics	Alprazolam	76 (4.7)
	Clonazepam	47 (2.9)
	Chlordiazepoxide	46 (2.8)
	Lorazepam	28 (1.70
	Diazepam	16 (1)
	Oxazepam	13 (0.8)
	Flurazepam	1 (0.06)
	Zolpidem	3 (0.18)
	Total	227 (14)
Cardio-vascular	Digoxin	30 (1.8)
	Prazosin	13 (0.8)
	Terazosin	8 (0.5)
	Methyldopa	2 (0.12)
	Amiodarone	1 (0.06)
	Dipyridamole	1 (0.06)
	Ticlopidine	1 (0.06)
	Total	56 (0.46)
Gastro-intestinal	Proton pump inhibitors	91 (5.6)
	Clidinium C	87 (5.4)
	Metoclopramide	2 (0.12)
	Dicyclomine	1 (0.06)
	Total	181 (11.18)
Others	Glibenclamide	228 (14.25)
	Phenobarbital	7 (0.43)
	Trihexyphenidyl	6 (0.37)
	Dimenhydrinate	6 (0.37)
	Hydroxyzine	5 (0.31)
	Methocarbamol	4 (0.25)
	Diphenhydramine	3 (0.18)
	Ergotamine	3 (0.18)

According to table 4, there was a significant difference between gender, underlying disease, life alone and polypharmacy and inappropriate drugs (p<0.05). No significant differences were detected between the level of education, age, insurance and marriage. Polypharmacy and underlying disease were the most frequent (table 4).

**Table 3:** Relationship between some variables and drug interactions, in prescriptions for elderly patients (Cohort study of Amirkola, AHAP, 2011-2012)

**Variable**	**Subgroup**	**Frequency (%)**	**P-value**	**Unadjusted OR (95%CI)**
Age	60-64	173 (30.1)	0.439	0.99 (0.983, 1.014)
	65-69	117 (34.9)		
	70-74	107 (37.8)		
	75-79	66 (26)		
	≥80	49 (28.8)		
Sex	Male	233 (26.4)	0.001	1.714(1.388, 2.118)
	Female	279 (38.1)		
Underlying Disease	Yes	511 (35.6)	0.001	100.21 (13.998, 717.360)
	No	1 (0.5)		
Education	Illiterate	309 (29.6)	0.42	1.21 (0.948, 1.544)
	Elementary	160 (34.4)		
	Diploma and above	43 (40..6)		
Living with	Family	467 (31)	0.028	1.59 (1.070, 2.370)
	Alone	45 (41.7)		
Marriage	Married	415 (30.1)	0.001	1.60 (1.203, 2.119)
	Single	97 (40.8)		
Insurance	Yes	456 (34.1)	0.0001	2.05 (1.497, 2.806)
	No	56 (20.1)		
Polypharmacy	Yes	294 (78.6)	0.0001	17.26 (12.954, 23.004)
	No	218 (17.6)		

**Table 4:** Relationship between some variables and inappropriate drugs, in prescriptions for elderly patients (Cohort study of Amirkola, AHAP, 2011-2012)

**Variable**	**Subgroup**	**Number (%)**	**P-value**	**Unadjusted OR (95%CI)**
Age	60-64	229 (39.9))	0.730	0.998 (0.981, 1.015)
	65-69	127 (37.9)		
	70-74	116 (41)		
	75-79	99 (39)		
	≥80	75 (44.1)		
Sex	Male	285 (32.3)	0.0001	2.036 (1.663, 2.492)
	Female	361 (49.2)		
Underlying Disease	Yes	625 (43.6)	0.0001	5.923 (3.715, 9.444)
	No	21 (11.5)		
Education	Illiterate	416 (39.8)	0.527	1.313 (1.009, 1.707)
	Elementary	197 (42.4)		
	Diploma and above	33 (31.1)		
Living with	Family	592 (39.3)	0.036	1.547 (1.046, 2.288)
	Alone	54 (50)		
Marriage	Married	537 (39)	0.056	1.323 (1.003, 1.749)
	Single	109 (45.8)		
Insurance	Yes	531 (39.7)	0.650	0.933 (0.717, 1.213)
	No	115 (41.4)		
Polypharmacy	Yes	279 (74.6)	0.0001	7.002 (5.384, 9.107)

**Figure 1:**
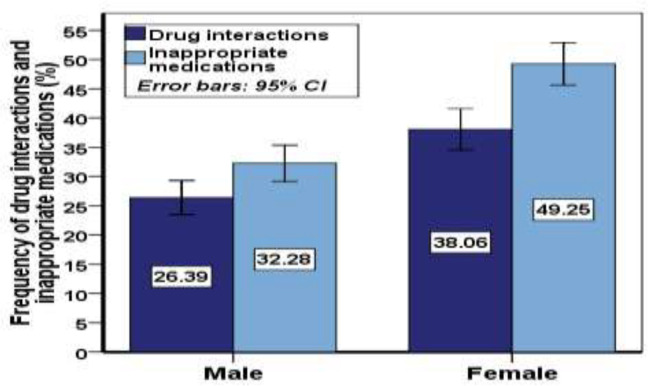
Comparison of drug interactions and inappropriate medications based on Beers criteria by sex (Cohort study of Amirkola, AHAP, 2011-2012)

**Figure 2.**
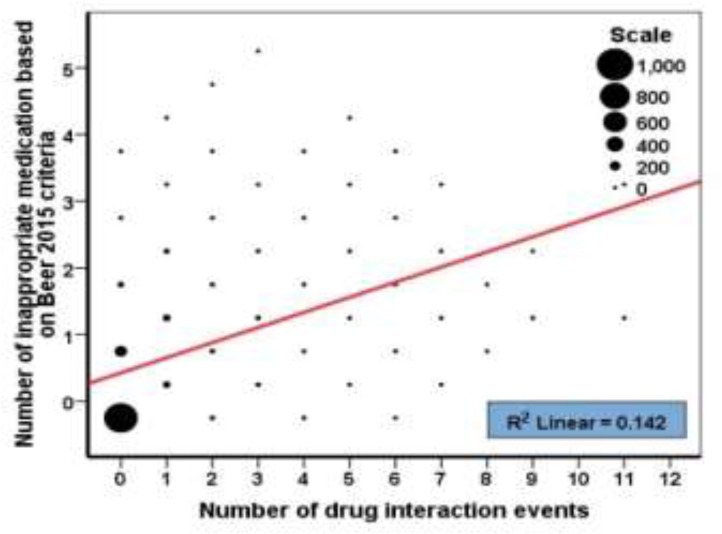
Correlation between number of drug interaction events and number of inappropriate medication based on Beers 2015 criteria (Cohort study of Amirkola, AHAP, 2011-2012)

**Figure 3:**
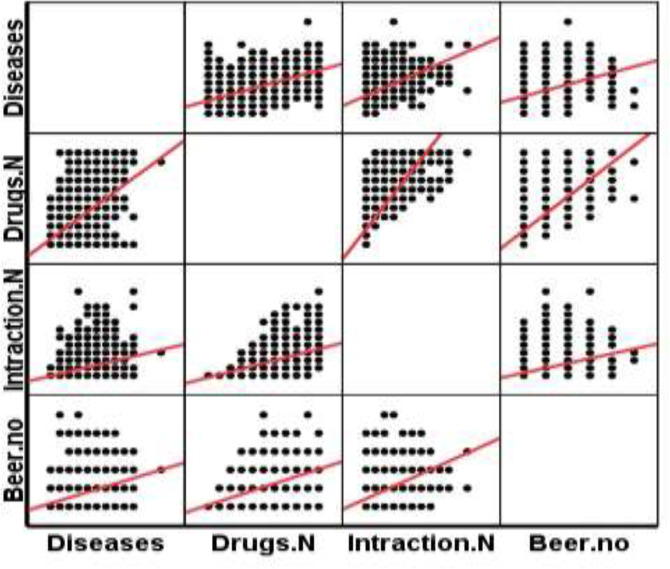
Correlation between drug interaction events and underlying diseases based on Beers 2015 criteria [Drug.N: drug number, Interaction.N: interaction number. (Cohort study of Amirkola, AHAP, 2011-2012)]

According to figure 1, the probability of drug interactions was higher in females [38.06, 95% CI; 34.53, 41.58] than in males [26.387, 95% CI; 23.47, 29.29], (p<0.05). The rate of inappropriate medication [49.24, 95% CI; 45.62, 52.87] in women was higher than men [32.27, 95% CI; 29.18, 35.36[, (p<0.05). According to table 5, among the drug categories, cardiovascular drugs accounted for a higher percentage of the drug interactions (frequency: 440), which also showed a significant difference (table 5). Subsequently, pain killers also significantly increase the likelihood of drug interactions in patients (frequency: 411). As shown in figure 2, there is a low correlation between drug interactions and inappropriate, as inappropriate medication may not cause drug interaction (figure b2). There is a significant correlation between drug interactions with polypharmacy and underlying disease. Underlying disease has a significant correlation with polypharmacy, but underlying disease and polypharmacy are not correlated with inappropriate medication (figure 3). 

**Table 5:** Frequency (%) of drug interactions and inappropriate medications based on Beers 2015 criteria, in prescriptions for elderly patients (Cohort study of Amirkola, AHAP, 2011-2012)

**Drug Group**	**Drug Interaction**	**P- value**	**Unadjusted OR** **(95% CI)**	**Inappropriate Drug**	**P -value**	**Unadjusted OR** **(95% CI)**
Analgesics	411 (65.6)	0.0001	16.729 (12.853, 21.775)	408 (65.1)	0.0001	5.879 (4.720, 7.322)
Gastrointestinal	158 (50.5)	0.0001	2.733 (2.122, 3.519)	254 (81.2)	0.0001	10.050 (7.360, 13.600)
Anti hyperlipidemia	221 (73.7)	0.0001	9.854 (7.388, 13.143)	179 (59.7)	0.0001	2.689 (2.080, 3.477)
Benzodiazepines	130 (61.30)	0.0001	32.773 (18.493, 58.079)	199 (93.9)	0.0001	32.773 (18.493, 85.079)
Antidiabetics	189 (67)	0.0001	6.361 (4.817, 8.400)	244 (86.5)	0.0001	14.887 (10.373, 21.364)
Respiratory Drugs	22 (34.9)	NS^*^	1.164 (0.686, 1.976)	32 (50.8)	NS	1.579 (0.953, 2.614)
Neurologic Drugs	114 (55.9)	0.0001	.227 (2.391, 4.355)	149 (73)	0.0001	4.688 (3.593, 6.923)
Supplementary Drugs	108 (46.4)	0.0001	2.094 (1.579, 2.777)	139 (59.7)	0.0001	2.555 (1.924, 3.393)
Cardiovascular Drugs	440 (64.4)	0.0001	21.653 (16.251, 28.850)	392 (57.4)	0.0001	3.601 (2.921, 4.439)
Hormonal Drugs	16 (32)	NS	1.015 (0.555, 1.857)	23 (46)	NS	1.289 (0.733, 2.269)
Antibiotics	11 (40.7)	NS	1.493 (0.688, 3.240)	16(59.3)	NS	2.214 (1.021, 4.802)
Antihistaminic Drugs	9 (50)	NS	2.177 (0.859, 5.517)	14 (77.8)	NS	5.350 (1.753, 16.326)
Kidney Drugs	8 (42.1)	NS	1.577 (0.631, 3.645)	11 (57.9)	NS	2.083 (0.83, 5.207)
Eye Drugs	8 (38.1)	NS	1.332 (0.549, 3.234)	13 (61.9)	NS	2.470 (1.018, 5.992)
Topical Drugs	0	NS	0.683 (0.661, 0.706)	0	NS	0.399 (0.376, 0.424)

* NS: not-significant

## Discussion

The growing elderly population has created many problems in numerous aspects of health. Elderly people use various drugs in large numbers due to physiological changes and some accompanying diseases. For this reason, multiple drug use and physical problems increase the probability of drug events such as drug interactions or adverse drug reactions. In the present study, the rate of drug interactions in the elderly was 31.7%, of which 87% were moderate and 12% were severe. However, in the study of Khoury et al. (2006) on drug interactions in physicians prescription in Gorgan, drug interaction was reported about 8.3% (87% of which was severe drug interaction) (15).

Additionally, in the cross-sectional study of Bogetti et al. (2016) in Mexico on 181 elderly patients with dementia, 59.1% had potential drug interactions and 59.81% of which had severe drug interactions. The most common drug interactions were reported in the combination of citalopram-antiplatelet drugs (11.6%), clopidogrel-omeprazole (6.1%) and clopidogrel-aspirin (5.5%) (7). In our study, the combination of aspirin-enalapril and atenolol-propranolol each had a rate of 26.2% of the most common severe drug interactions, indicating that prescribing medications in the elderly requires careful attention to avoid severe interactions.

According to the Beers 2015 Criteria in our study, 446 drugs were found to be inappropriate for the elderly. Of these, the most frequent were NSAIDs (245, 15%). But in the Binit’s study, according to the Beers 2012 criteria, at least one inappropriate drug was found in 87.3% of cases. Metoclopramide, alprazolam, diazepam, digoxin and diclofenac were the most common prescription drugs. NSAIDs, like the present study in cardiovascular patients, were common drugs involved in drug complications and drug interactions in the elderly (9). 

Cardiovascular drugs and painkillers were the most common drugs that significantly increased the probability of drug interactions in the elderly. On the other hand, the use of topical drugs was less likely to interfere with other medications. However, in the study of Khoury et al., cholinergic drugs with furazolidone, clonidine with tricyclic antidepressants, and penicillin with tetracycline, respectively, were the most common drugs that showed interactions (15). 

Variables such as gender, age, literacy, occupation, living alone or with family, marital status, underlying disease and polypharmacy were studied in this study. In women, living alone, having underlying disease, polypharmacy and having insurance, were significantly correlated with drug interaction vents and inappropriate medication levels according. In agreement with our study, Lima et al. (2009) reported that there is a significant difference in drug interactions between men and women, which can be justified by the fact that women are referred to physicians higher and therefore take more drugs (16).

In the study of Ahmadi et al. (2006), the polypharmacy and consequently drug interactions were higher in the age group of 65-74 years and in those with primary and lower education level (17). However, in our study, most of the interactions (37.8%) were seen in the age group of 74-70 years, although no significant difference was observed. On the other hand, a higher percentage of people with high school and university education (40.6%) have drug interactions than illiterate people (29.6%). It can be argued that people with higher education may use more self-medication. Although there is generally no significant difference between them, it can be justified by this hypothesis that patients are likely to have no role in the choice and administration of their drugs, and physicians select and prescribe medications. The present study showed that the occurrence of drug interactions is directly correlated with the number of drugs prescribed. These results are consistent with the study by Nazarian et al. (2010) in Tehran, which showed that more polypharmacy causes more likely of drug interactions (18). Moghadamnia et al. (2002) also reported that general practitioners prescribe unnecessary drugs such as antibiotics and NSAIDs for patients which may increase occurrence of drug interactions (19). They suggested educating drug information to medical students and physicians properly can reduce over-prescribing and subsequently may reduce drug interactions (19).

The results of the present study indicate that drug interaction and having underlying disease have a significant relationship with each other, as the underlying disease obviously leads to more drug use. As previously reported in the Bahat’s study (2013), several drugs with multiple underlying diseases such as cardiovascular disease, diabetes, and stroke usually require multiple medications for their treatment simultaneously. This may increase drug events such as drug interaction (20). 

According to the results of our study, it is remarkable that patients with at least one type of insurance significantly showed more likely drug interactions. It is concluded that patients are more likely to see a doctor and receive more drugs because of the cheap insurance covered drugs. There was a significant difference between living alone and living with family, which explains that elderly living alone may be less careful about taking different drugs. On the other hand, they may also take non-prescription and inappropriate drugs as self-medications. According to the results of simultaneous logistic regression, gender and polypharmacy variables and underlying diseases were the influencing variables on drug interactions. Whereas, in this analysis, the omission of the polypharmacy variable reduced the effect of the gender variable, but the omission of the polypharmacy variable associated with the underlying diseases again made the gender variable significant. Therefore, it is suggested that women are more likely to take more medications due to their referral to physicians and their diagnosis of underlying diseases, which increases the likelihood of drug interactions.

The limitations in this study were lack of cooperation of some patients, recall bias and/or inability to recall the kind and number of their given drugs, self-medication by the subjects, weak knowledge for the used drugs and possible death of some participants in next visit. However, the strengths of this study were high participation rate (72%), being a population based-study and observing and reviewing all medications for the patients.

In this study, the rate of drug interactions in the prescription of the elderly was 31.7%, which is higher than in other studies. For this reason, the right prescribed medications by doctors are very important for the elderly. On the other hand, the relationship between polypharmacy and underlying disease with drug interaction indicates that care should be taken in prescribing multiple drugs in the elderly, especially with various underlying diseases.
